# Reproducibility of MRI Radiomics Measurements in Men with Prostate Cancer Undergoing Active Surveillance

**DOI:** 10.3390/cancers18050778

**Published:** 2026-02-28

**Authors:** Himanshu Sharma, Haitham Al-Mubarak, Juan Lloret Del Hoyo, Ghadi Abboud, Octavia Bane, Mickael Tordjman, Mira M. Liu, Vinayak Wagaskar, Ashutosh Tewari, Bachir Taouli, Sara Lewis

**Affiliations:** 1 Department of Diagnostic, Molecular and Interventional Radiology, Icahn School of Medicine at Mount Sinai, One Gustave L. Levy Place, New York, NY 10029, USA; 2 Biomedical Engineering and Imaging Institute, Icahn School of Medicine at Mount Sinai, One Gustave L. Levy Place, New York, NY 10029, USA; 3 The Department of Urology, Icahn School of Medicine at Mount Sinai, One Gustave L. Levy Place, New York, NY 10029, USA

**Keywords:** prostatic neoplasms, magnetic resonance imaging, radiomics, reproducibility

## Abstract

**Background**: MRI-based radiomics has shown promise in men with prostate cancer (PCa); however, successful clinical implementation is contingent upon on reproducible measurements. **Purpose**: We assessed the reproducibility of radiomics features extracted from bi-parametric prostate MRI (bpMRI) in prostate lesions and non-tumoral prostate tissue in men with PCa undergoing active surveillance (AS). **Methods**: This retrospective study included 47 men with biopsy-proven PCa undergoing AS (mean 68.9 ± 8.2 years, mean PSA density [PSAD] 0.08 ± 0.03 ng/mL/mL) who underwent two bpMRI approximately 12 months apart (range, 10–14 months; December 2018 to April 2020). The reproducibility of radiomics measurements was assessed using the same MRI platform (3T Skyra, Siemens Healthineers; inter-platform) (n = 37), different MRI vendors (Skyra, Siemens Healthineers; 3T Discovery MR750, GE Healthcare; inter-platform) (n = 10), and between observers (n = 10). Shape/1st-/2nd-order radiomics features were extracted from regions of interest on axial T2-weighted (T2-WI), diffusion-weighted imaging (DWI, b1600), and apparent diffusion coefficient (ADC) maps on prostate lesions, non-tumoral peripheral zones (PZs), and transition zones (TZs) using software. Reproducibility was evaluated by calculating the intraclass correlation coefficient (ICC) and coefficient of variation (CV). Associations of clinical variables and prostate volume were assessed. **Results**: PCa diagnoses included Gleason grade groups 1 (n = 46) and 2 (n = 1)]. Thirty-seven lesions (mean size 0.9 ± 0.4 cm) in 31 patients had PI-RADS v2.1 scores of 2 (n = 3)/3 (n = 12)/4 (n = 21)/5 (n = 1); 16 patients demonstrated diffuse PI-RADS 2 changes. Lesion radiomics features from T2-WI yielded a high proportion of good/moderate ICCs (intra-platform, 77.8%; inter-platform, 56.5%), whereas most DWI/ADC features yielded poor reproducibility. Similar results were observed for non-tumoral PZ/TZ. Intra-platform CVs were lowest for T2-WI lesion features (13.6%) and background PZ/TZ (<13.3%), while DWI/ADC exceeded 20%. Inter-platform CVs were lowest for lesions on T2-WI and were <18% for DWI/ADC; all background PZ/TZ CVs were < 16.4%. Inter-observer analyses showed good/moderate ICCs across all sequences and regions (57.4–92.6%). The distribution of ICC and CV values did not differ between intra- and inter-platform analyses (*p* > 0.05). Higher reproducibility (ICC > 0.5) was associated with larger prostate volume (intra-platform diagnostic odds ratio [DOR] = 2.58, 95% confidence interval [95%CI], 1.35–3.80, *p* = 0.01; inter-platform DOR = 3.48, 95%CI 1.79–5.17, *p* = 0.01) and older age (inter-platform DOR = 5.30, 95%CI 3.75–6.85, *p* < 0.01). **Conclusions**: Radiomics measurements from T2-WI demonstrated better intra-/inter-platform reproducibility than DWI/ADC for prostate lesions and non-tumoral tissue. Patient factors (larger prostate volumes and older age) influence radiomics stability. The optimization of diffusion-based radiomics features is needed to improve reproducibility given the essential role of DWI in prostate MRI.

## Simple Summary

This retrospective study evaluated the reproducibility of MRI-based radiomics features extracted from bi-parametric prostate MRI in 47 men with biopsy-proven prostate cancer undergoing active surveillance. The subjects underwent two MRI exams approximately 12 months apart, allowing for assessment of radiomics stability across repeated scans. Reproducibility was analyzed using the same MRI platform (intra-platform), different MRI platforms (inter-platform), and between observers. Radiomics features were extracted from prostate lesions and non-tumoral peripheral and transition zones on T2-weighted imaging (T2-WI), diffusion-weighted imaging (DWI), and apparent diffusion coefficient (ADC) maps. T2-WI radiomics features demonstrated the highest reproducibility, showing the greatest proportion of moderate to good intraclass correlation coefficients and the lowest coefficients of variation. However, DWI and ADC-based features showed poorer stability. Inter-observer agreement was generally moderate to high across sequences. Larger prostate volume and older age were associated with improved radiomics reproducibility. These findings highlight the need to optimize diffusion-based radiomics to support reliable clinical implementation.

## 1. Introduction

Prostate cancer (PCa) is the second leading cause of cancer-related death among men in the United States [1]. Active surveillance (AS) is a conservative management approach for the monitoring of patients with low-risk or favorable intermediate-risk PCa to either avoid overtreatment in men with indolent disease or to defer treatment until progression of disease is observed [2,3]. Guidelines for AS eligibility vary, but inclusion criteria typically include clinical stage, life expectancy, prostate-specific antigen (PSA), and Gleason score [4]. Prostate MRI is an increasingly attractive alternative method for PCa diagnosis and disease monitoring [5,6,7,8,9,10]. Prostate MRI combines T2-weighted (T2-WI) imaging with one or more sequences such as diffusion-weighted imaging (DWI)/apparent diffusion coefficient (ADC) [termed bi-parametric (bp)MRI] or T2-WI, DWI and dynamic contrast-enhanced MRI (DCE)-MRI [termed multiparametric (mp)MRI] [11]. The standardization of terminology and qualitative guidelines for imaging assessment using Prostate Imaging Reporting and Data System version 2.1 (PI-RADS v2.1) improved the reproducibility and accuracy of MRI [8,12]. While MRI and PI-RADS scoring has dramatically changed the diagnostic and management pathways in PCa, there remains an urgent need to have repeatable and reproducible noninvasive imaging markers of tissue and tumor status.

Radiomics techniques extract quantitative features from imaging studies that can be used for a variety of clinical tasks [13]. Numerous studies have investigated the utility of radiomics in the detection of PCa and differentiating indolent and aggressive tumors [14,15,16,17]. Radiomics may be a useful tool in predicting the behavior of high-grade PCa and has been used to risk stratify patients according to the likelihood of increasing the Gleason grade group (GGG) [14,18,19,20]. Furthermore, the use of radiomics features has been shown to improve PI-RADS performance [21]. While radiomics has been shown to be a promising tool in the imaging of PCa, the clinical utility of radiomics partially depends on the establishment of its reproducibility. Multiple potential sources of measurement variability can impact the utility of radiomics, including MRI platform, field strength, and scanning protocol, which has been shown in other abdominopelvic malignancies (rectal, cervical, liver cancer) and tissues (liver parenchyma) [22,23,24,25]. Prior work has found that some radiomics features demonstrate repeatability with a high intraclass correlation coefficient (ICC) in a small set of patients who underwent test–retest mpMRI of the prostate within two weeks, although data are limited [26,27]. Further, DWI with ADC quantification is the most important sequence in prostate MRI, for driving PI-RADS classification for peripheral zone lesions and as a quantitative marker reflecting tissue cellularity and microstructure [12,28]. However, ADC measurements exhibit substantial variability, limiting comparability among study populations [29,30]. Knowledge of the repeatability of radiomics in men with prostate cancer undergoing active surveillance is needed to aid the clinical translation of radiomics as a noninvasive marker or tumor biology. The purpose of this study is to assess the reproducibility of radiomics features extracted from bi-parametric prostate MRI (bpMRI) in prostate lesions, the non-tumoral peripheral zone (PZ), and the transition zone (TZ) in men with clinically stable low-grade prostate cancer undergoing AS.

## 2. Methods and Materials

### 2.1. Patient Selection

**Figure 1:**
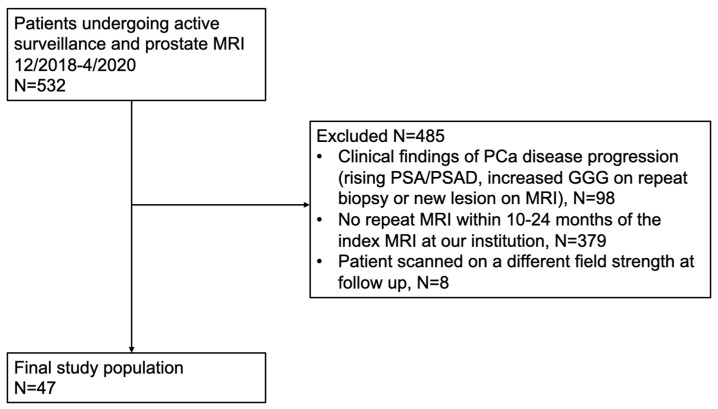
Study flow chart is summarized.

The Institutional Review Board at the Icahn School of Medicine at Mount Sinai (#Study-21-01490) approved this retrospective single-center study with waiver of informed consent on 15 December 2021. A waiver of informed consent was justified for this retrospective study because it involves minimal risk to participants, using existing data collected for clinical purposes. We queried the clinical database of men with low or favorable intermediate-risk PCa undergoing active surveillance (AS), in whom the decision to pursue AS was jointly determined by the clinician and the patient based on clinical, laboratory, and pathologic data, who underwent two mpMRIs between December 2018 and April 2020. AS is an established management option for patients with low-risk and favorable intermediate-risk PCa [3]. This time period was selected as the MRI protocol, and sequence parameters were not modified during this time at our institution. The time interval of approximately 12 months was selected as biologically meaningful intervals relevant to prostate cancer progression and is commonly used in clinical practice. Inclusion criteria included men in whom the biopsy-confirmed PCa was deemed stable over the study period by the clinician based on clinical, laboratory or pathologic data and underwent repeat MRI approximately one year after the index MRI. Exclusion criteria for our study were men with PCa progression (rising PSA/PSAD, increased Gleason grade group (GGG) on repeat biopsy, and patients with new or worsening lesions on second mpMRI, n = 98), men who did not have repeat MRI at our institution during the follow-up period (n = 379), or men who underwent MRI at a different field strength (n = 8). Forty-seven patients with PCa, thus, met inclusion criteria for this study (Figure 1). Patient demographics, including age, race and ethnicity, laboratory values, and biopsy data were extracted from the electronic medical record by the study coordinator (Observer 1, a fourth-year medical student).

### 2.2. MRI Protocol

All patients underwent two bpMRIs of the prostate using a 3T MRI platform (Skyra, Siemens Heathlineers, Erlangen, Germany; Discovery MR750, GE Healthcare, Chicago, IL, USA) over approximately 12 months (range, 10–14 months) as per clinical protocol between December 2018 and April 2020. The MRI protocol and acquisition parameters remained stable during the study period (Supplemental Table S1). The MRI protocol included T2-WI, DWI (b-values of 50, 1000, 1500–1600) and apparent diffusion coefficient (ADC). ADC calculation was based on mono-exponential fitting of the DWI signal on a pixel-by-pixel basis in line on the scanner [31]. Data regarding the presence and size of lesions and PI-RADS v2.1 scores were extracted from the clinical report by the study coordinator (Observer 1) and verified by a senior radiologist with expertise in prostate imaging (Observer 2, with 12 years of experience).

### 2.3. Image Analysis

#### 2.3.1. Prostate and Tumor Segmentation

One observer (Observer 3, with 1 year of expertise in prostate imaging) manually placed circular regions of interest (ROIs) on the peripheral zone (PZ), transition zone (TZ) at the base, midgland, and apex of the prostate gland and on prostate lesions reported on the MRI (≥PI-RADS 2), if present, on T2-WI, high b-value DWI and ADC for both the baseline and follow-up MRIs. Prostate lesion boundaries were carefully prescribed to remain within the lesion and avoid surrounding non-tumoral structures, including peritumoral edema. No necrotic regions were present, as none of the lesions had undergone prior treatment. Inter-observer analysis was performed in 10 randomly selected patients in the study population by a second observer who was blinded to the first observer’s segmentations (Observer 4, with 1 year of experience in prostate imaging). Each observer was blinded to each other’s segmentations. Examples of ROI segmentations are shown in Supplementary Figure S1. All ROIs were prescribed using software compliant with the Image Biomarker Standardization Initiative (IBSI) guidelines (Olea sphere^®^ 3.0, Olea Medical, La Ciotat, France).

#### 2.3.2. Image Pre-Processing and Radiomics Feature Extraction

The IBSI established a framework for standardized computational practices and widely adopted image-processing techniques for radiomic feature extraction, all of which were adhered to in the present study [32]. Spatial resampling was performed using nearest neighbor interpolation to create isotropic voxels (1.0 × 1.0 × 1.0 mm^3^) to allow comparison between image data. Signal intensity normalization was performed on T2-WI and DWI at b1500–1600. No normalization approach was performed on ADC as it reflects a quantitative property. A 64-fixed bin number was used for intensity discretization, as per Image Biomarker Standardization Initiative (IBSI) guidelines [32]. Shape (n = 16), first-order (histogram, n = 19), and second-order (texture, n = 73) radiomics features were extracted. The texture feature classes included Gray-Level Co-Occurrence Matrix (GLCM, n = 23), Gray-Level Run-Length Matrix (GLRLM, n = 16), Gray-Level Size Zone (GLSZM, n = 15), Gray Tone Difference Matrix (GLDM, n = 13) and Neighboring Gray Tone Difference Matrix (NGTDM, n = 6).

### 2.4. Statistical Analysis

Descriptive statistics were used to summarize the characteristics of the study population. For test–retest, inter-platform and inter-observer reproducibility, we used the intraclass correlation coefficient (ICC, the degree of absolute agreement among measurements), which is commonly used in radiomics studies and remains unaffected by linear scaling or shifting [26,33]. ICC for reporting test–retest repeatability and inter-platform and inter-observer reproducibility were classified as follows: good (ICC ≥ 0.8); moderate (0.5–0.8); or poor (<0.5). Results for each group of radiomics features (shape, 1st and 2nd order) within PZ and TZ ROIs and lesion ROIs for each MRI sequence were reported as median ICC. The coefficient of variation (CV), defined as the ratio of the standard deviation to the mean of measurements, was also analyzed to evaluate the reproducibility of radiomic measurements for lesions and background tissue across all sequences within (intra-platform) and across imaging platforms (inter-platform), with median and interquartile range values reported. CV measurements were classified as follows: excellent (CV ≤ 10%), good (11–20%), moderate (21–30%), and poor (>30%) [34,35]. The difference in distributions of CV and ICC between intraplatform and inter-platform analyses was calculated by comparing Student’s *t*-test *p* < 0.05 and Mann–Whitney U-test *p* < 0.05. The effect of clinical demographics (age, body mass index [BMI], and PSAD) and prostate volume on MRI on ICC values was examined with univariate cross-validated logistic regression. Models were built to test the ability to predict an ICC over 0.5 solely from age, prostate volume, and PSA density. We considered as statistically significant a *p*-value < 0.05. All the statistical analyses were calculated using MATLAB 2024b software.

## 3. Results

### 3.1. Patients

The demographic and clinical characteristics of the patient population are summarized in Table 1. In 47 men (mean age 68.9 ± 8.2 y), the mean PSA was 4.4 ± 2.5 and the PSA density (PSAD) was 0.08 ± 0.03 ng/mL^2^. Prostate biopsy revealed Gleason grade group (GGG) 1 in 46 patients and GGG 2 in 1 patient. Thirty-seven lesions were identified in 31/47 patients at the first MRI and were included in this study, with the following PI-RADSv2.1 scores: 2 (n = 3); 3 (n = 12); 4 (n = 21); and 5 (n = 1). Mean lesion size at initial MRI was 0.86 ± 0.37 cm. At the second MRI, the following distribution of PI-RADSv2.1 scores was observed: 2 (n = 5); 3 (n = 12); 4 (n = 19); and 5 (n = 1). Two lesions were reclassified based on the PI-RADS score at the follow-up MRI from PI-RADS 4 to PI-RADS 2. The remaining patients had PI-RADS 2 changes reported in the peripheral zone, and no discrete lesion was identified.

### 3.2. Intra-Platform (Test-Retest) Reproducibility

**Figure 2:**
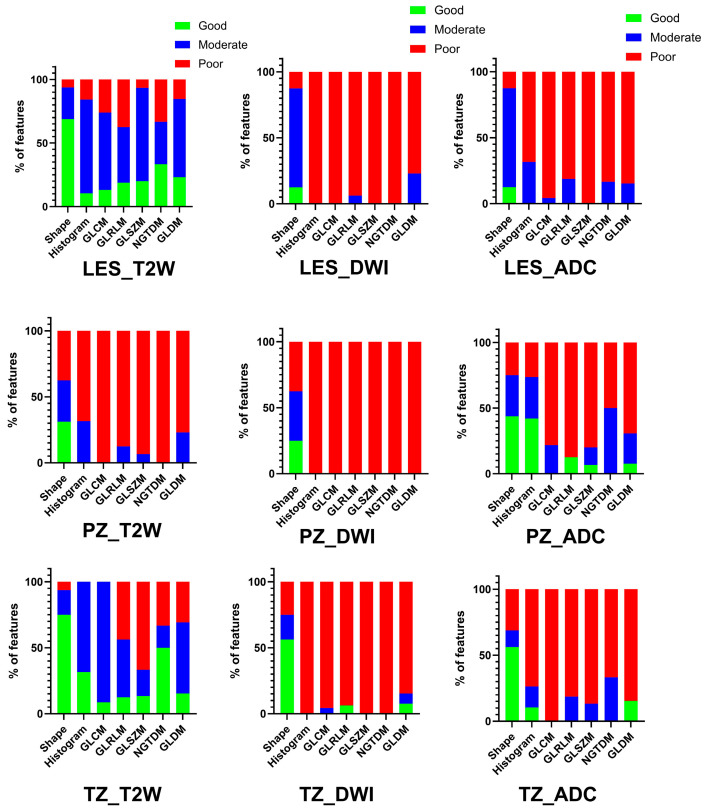
Intra-platform (test–retest) reproducibility [prostate lesions, top row; background peripheral zone (PZ), middle row; background transition zone (TZ), bottom row] of radiomic features extracted from T2-WI MRI had greater proportion of features with moderate/good intraclass correlation coefficients (ICCs), whereas mostly poor ICCs were found for radiomics measurements from DWI and ADC. Abbreviations: PZ (peripheral zone); TZ (transitional zone); T2W (T2-weighted imaging); DWI (diffusion-weighted imaging); ADC (apparent diffusion coefficient); GLCM (Gray-Level Co-Occurrence Matrix); GLRLM (Gray-Level Run-Length Matrix); GLSZM (Gray-Level Size Zone); GLDM (Gray Tone Difference Matrix); NGTDM (Neighboring Gray Tone Difference Matrix).

For patients imaged on the same MRI platform for both exams (n = 37), T2-WI was found to have the highest proportion of good/moderate radiomics feature measurement repeatability (77.6% for lesions, 47.1% for PZ, and 81.4% for TZ). DWI (18.4% for lesions, 12.9% PZ, and 20.3% TZ) and ADC (25.8% for lesions, 37.0% PZ, and 44.3% TZ) had a lower proportion of feature repeatability (Figure 2). Across all radiomics feature classes, low CV (13.6%) was found for measurements from lesions on T2-WI, while DWI and ADC showed high CoV (>20%). The CVs for radiomics measurements for the background TZ and PZ were all less than 13.3%. These results are summarized in Table 2.

### 3.3. Inter-Platform Reproducibility

**Figure 3:**
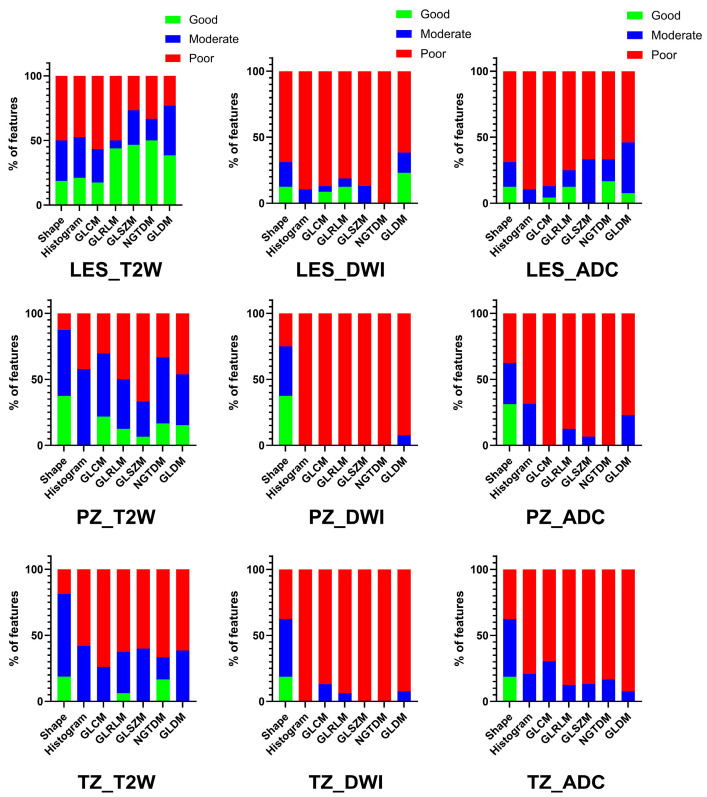
Inter-platform assessment performed [prostate lesions, top row; background peripheral zone (PZ), middle row; background transition zone (TZ), bottom row] of radiomic features extracted from T2-WI MRI also had greater proportion of features with moderate/good intraclass correlation coefficients (ICCs), whereas mostly poor ICCs were found for radiomics measurements from DWI and ADC. Abbreviations: PZ (peripheral zone); TZ (transitional zone); T2W (T2-weighted imaging); DWI (diffusion-weighted imaging); ADC (apparent diffusion coefficient); GLCM (Gray-Level Co-Occurrence Matrix); GLRLM (Gray-Level Run-Length Matrix); GLSZM (Gray-Level Size Zone); GLDM (Gray Tone Difference Matrix); NGTDM (Neighboring Gray Tone Difference Matrix).

Similar results were found for patients scanned on different MRI platforms/same field strength (n = 10), as T2-WI was found to have the highest proportion of good/moderate measurement repeatability (56.4% for lesions, 60.1% for PZ, and 42.5% for TZ). DWI (18.4% for lesions, 11.9% for PZ, and 13.8% for TZ) and ADC (24.9% for lesions, 24.0% PZ, and 24.9% for TZ) had a lower proportion of feature repeatability (Figure 3). The CVs of radiomics measurements across all features for lesions, DWI and ADC were 10.7%, 17.8% and 18.0%, respectively. The CVs of radiomics measurements were all less than 16.4% for background PZ and TZ for all sequences. Table 3 summarizes these results.

### 3.4. Distribution of Radiomics Measurements Between Intra-/Inter-Platform Analyses

**Figure 4:**
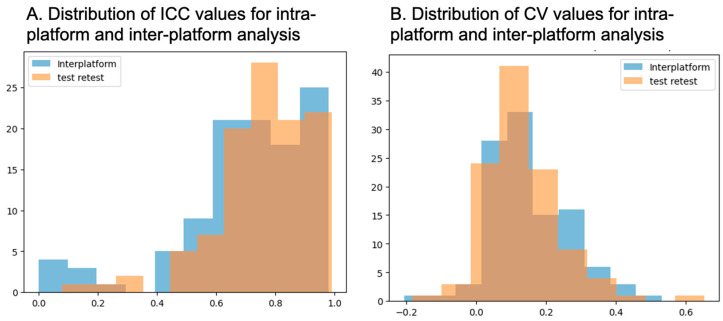
The histogram distribution of ICC values from the intra-platform (test–retest) analysis of radiomic measurements obtained from prostate lesions and background tissue on T2-WI, DWI and ADC shows higher mean and median ICC values, along with a smaller interquartile range (IQR), compared to the inter-platform analysis (**A**). Similarly, the intra-platform (test–retest) coefficient of variation (CV) of the same radiomics measurements exhibits lower mean and median CV values, as well as a smaller IQR, compared to the inter-platform analysis (**B**). However, these differences were not statistically significant (all *p*-values >0.09), and there was overlap in the distribution of values between analyses.

The histogram distribution of ICC values from the intra-platform (test–retest) analysis of radiomics measurements obtained from prostate lesions and background tissue on T2-WI, DWI and ADC shows higher mean and median ICC values, along with a smaller interquartile range (IQR), compared to the inter-platform analysis. Similarly, the intra-platform (test–retest) coefficient of variation (CV) of the same radiomics measurements exhibits lower median CV values, as well as a smaller IQR, compared to the inter-platform analysis. However, there was no statistically significant difference in the distribution of radiomics reproducibility measures between intra-platform and inter-platform analyses (all *p*-values > 0.09), indicating the robustness of radiomics measurements, independent of MRI platform. Histogram maps of the distribution of the median ICC and CV values for the intra-platform and inter-platform comparisons of radiomics measurements demonstrate overlap (Figure 4).

### 3.5. Inter-Observer Reproducibility

**Figure 5:**
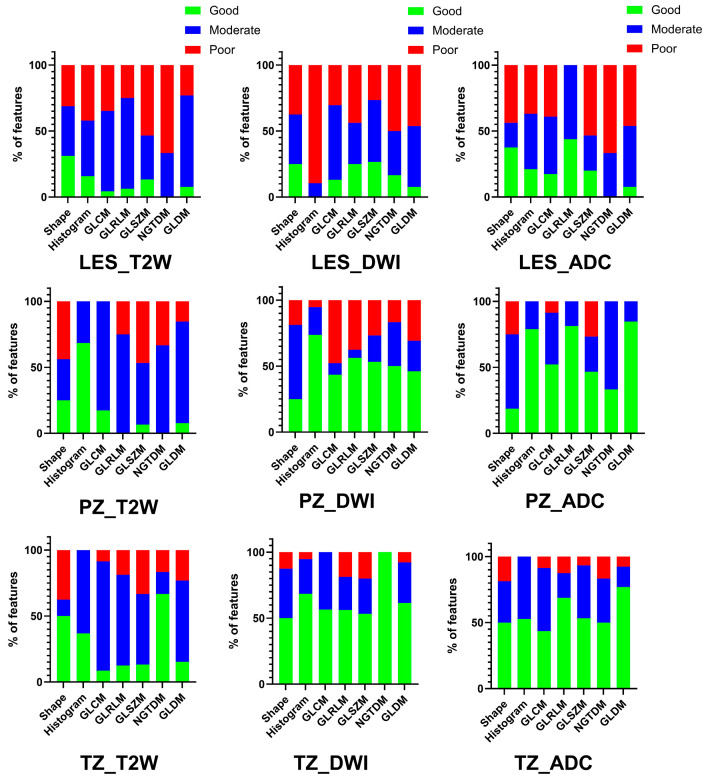
Inter-observer assessment of radiomic measurement reproducibility in prostate lesions and background PZ and TZ using interclass correlation coefficient (ICC). Overall, the majority of radiomics features yielded good/moderate ICCs (≥0.5) for all MRI sequences and regions. Abbreviations: PZ (peripheral zone); TZ (transitional zone); T2W (T2-weighted imaging); DWI (diffusion-weighted imaging); ADC (apparent diffusion coefficient); GLCM (Gray-Level Co-Occurrence Matrix); GLRLM (Gray-Level Run-Length Matrix); GLSZM (Gray-Level Size Zone); GLDM (Gray Tone Difference Matrix); NGTDM (Neighboring Gray Tone Difference Matrix).

Inter-observer analysis demonstrated that the majority of radiomics features yielded good/moderate ICCs for all sequences and regions (range, 57.4–92.6%) (Figure 5). These results are summarized in Table 4.

### 3.6. Effect of Clinical Variables and Prostate Volume on Radiomics Measurement Reproducibility

Logistic regression analysis found that larger prostate volume was associated with moderate/good ICC values (ICCs > 0.5) for both the intra-platform analysis (diagnostic odds radio [DOR] = 2.58, 95% confidence interval [CI] 1.35–3.80, *p* = 0.01) and inter-platform analysis (DOR = 3.48, 95%CI 1.79–5.17, *p* = 0.01). Patient age was associated with a higher rate of moderate/good ICC values for the inter-platform analysis (DOR= 5.30 95%CI 3.75–6.85, *p* < 0.01) but not at intra-platform analysis (*p* = 0.10). BMI and PSAD did not impact radiomic measurement reproducibility for either intra-platform (*p*-values 0.19 and 0.34, respectively) or inter-platform analyses (*p*-values 0.07 and 0.90, respectively). These results are summarized in Table 5.

## 4. Discussion

For radiomics to be validated as a marker of tumor or tissue biology in a clinical setting, knowledge of measurement reproducibility is essential and is currently not firmly established in prostate imaging. While initial studies have shown promise in the value of radiomics data from MRI in prostate cancer, the widespread adoption of the technique has been hindered by numerous factors. Multiple sources of variability can potentially impact the reproducibility of radiomics features in PCa, including the model of scanner used, field strength, acquisition parameters, post-processing software, observer, region of interest vs. volume of interest, patient-related factors, amongst others [36]. This study examined the reproducibility of radiomics features from bi-parametric MRI of the prostate in men with PCa undergoing active surveillance. The most important findings in our study were that radiomics measurements obtained from T2-WI demonstrated higher proportions of reproducible features compared to ADC and DWI for lesions as well as background PZ and TZ between a baseline MRI and follow-up exam approximately one year later in a population of men undergoing active surveillance (AS) for clinically stable PCa. Larger prostate volume (intra-/inter-platform analysis) and age (inter-platform analysis) were associated with a higher proportion of reproducible radiomics measurements but not body mass index (BMI) or PSA density (PSAD).

In a prior study, radiomics models were developed on bpMRI radiomics data in 459 men with clinical suspicion for PCa and visible lesions from one vendor and tested on a different vendor, which were found to be accurate and generalizable in distinguishing between benign and malignant lesions (mean AUC for the bpMRI model of 0.833), indicating the robustness of radiomics features between vendors [37]. In our study, we found that while there was overall variable reproducibility depending on the sequence, tissue or tumor being measured, and feature class, the distribution of ICC values for each sequence and tissue was not statistically different between the inter-platform and inter-platform analyses. The added value of our results includes our assessment of the background prostatic PZ and TZ, which can potentially serve as internal reference tissue. Further, MRI-invisible lesions are a common occurrence in men undergoing AS [38], as we observed in our study population. As expected, the inter-observer analysis found very good measurement reproducibility.

A prior study utilizing a publicly available dataset of 15 patients who underwent mpMRI and biopsy, and then underwent repeat mpMRI after 2 weeks, found 15 radiomics features on ADC (13 texture features, 2 shape and size features) and 18 radiomics features on T2-WI (17 texture features, 1 shape and size feature) that were highly reproducible [27]. However, another study using the same dataset found that, although there were a number of concordant features, radiomics features varied greatly in repeatability and were highly susceptible to variations in pre-processing settings [26]. For ADC images in this study, a number of features demonstrated ICCs > 0.7 in the tumor ROI, while for T2-WI, only the features Entropy, Energy, and Variance reached an ICC greater than the reference T2-W Volume ICC of 0.8 [26]. These findings contrast with our study, in which ADC showed relatively lower reproducibility, while T2-WI had the highest reproducibility of the sequences used. Our results in this study agree with the lower reproducibility for ADC/DWI radiomics analysis in renal masses, liver cancer and background liver parenchyma [22,39]. Possible explanations for the difference in our results include the sample size and technique (phased array coil in our study versus the use of endorectal coil in the prior study). The lower reproducibility of the radiomics features in ADC and DWI sequences may be caused by the lower spatial resolution in the DWI sequence, as well as the fact that DWI is acquired while free-breathing, introducing motion [22]. The lower spatial resolution results in larger voxel size, greater partial volume effects and image distortion from field inhomogeneity, gas, or motion, factors that impact radiomics feature measurement. The extent of the differences in radiomics measurements for DWI/ADC across the platforms and within the same platform was similar and is likely multifactorial, with minimal influence of b-value in this study. Given the differences in acquisition techniques, it is likely that the difference can be attributed to other sources than solely on b-value selection. However, further study on the DWI/ADC radiomics is warranted as ADC is widely recognized as a valuable marker of prostate cancer aggressiveness and is a key sequence for prostate lesion classification using PI-RADS [12,29].

Determining which radiomics feature class is the most reproducible is of value as well, given the large number of quantitative measures that can be extracted from available software packages. Histogram features generally demonstrated the highest ICCs for most analyses in our study, which is relevant as published studies have shown that these features have value for Gleason pattern classification [10,40] and distinction from background tissue [41]. A separate cross-site study of 406 voxel-wise radiomic features (gray, Haralick, gradient, Laws, and Gabor) found that over 99% of Haralick features were highly reproducible and achieved excellent tumor–non-tumor discrimination (≈0.8 accuracy) [42]. It is possible that second-order features are more influenced by differences in image resolution, signal-to-noise ratio, reconstruction parameters, and motion artifacts, more than other patient- or tumor-related factors, as compared to histograms [30].

Knowledge of how a patient’s clinical factors impact measurement reproducibility is highly relevant. The patient’s BMI and PSA density had no significant effect on radiomics measurement reproducibility, while larger prostate volume and older patient age (inter-platform only) improved reproducibility. Larger prostate glands occupy a larger portion of the field of view and are potentially less impacted by partial volume effects and have a greater number of voxels in the same anatomic zone (i.e., peripheral zone) [32]. Conversely, fewer voxels may be affected by the interface between glandular tissue and surrounding structures, which minimizes sensitivity to segmentation or registration variability with reduced noise. Older patients may show more reproducible radiomics features due, in part, to reductions in glandular heterogeneity over time decreasing the variability of radiomics features between scans, although we did not directly assess this.

This study has several limitations. Our data were gathered retrospectively from a single institution. This study had a modest sample size, with a smaller number of patients who were scanned inter-platform and a small number of patients with visible lesions. We did not assess the impact of normalization or image filtration on radiomics measurement robustness. The ranges of the follow-up scan varied from 8 to 14 months. While we excluded patients with significant changes in their clinical condition, relatively small variations in the underlying PCa not captured by the AS could have introduced variability into our results. However, our study meant investigated a clinical use case for radiomics in the setting of routine clinical variability in a real-world scenario.

## 5. Conclusions

The reproducibility of radiomics features for PCa has yet to be firmly established, currently limiting its utility as a clinical biomarker for men undergoing active surveillance. Establishing the generalizability of radiomics measurements across multiple sites and scanners is essential for the clinical adoption of methods. Our results demonstrate that radiomics measurements obtained from T2-W had a greater proportion of features with good/moderate ICC values, as compared to DWI/ADC. Certain clinical findings, such as BMI and PSAD, did not impact radiomics measurement reproducibility, while prostate volume and patient age did. Given the significant variability in MRI radiomics features in PCa in patients undergoing AS, features obtained from T2-WI, therefore, may be more useful in the longitudinal assessment of PCa in men undergoing AS. Further investigation into the reproducibility of radiomics features across sites (external validation), sequences, scanners, post-processing methods, and the application of deep learning-based or fully automated segmentation approaches, which could be used to minimize inter-observer variability and improve reproducibility, as well as clinical factors, is warranted.

## Figures and Tables

**Table 1:** Clinical, demographic, pathologic and imaging findings in study population (n = 47).

Parameter	Value	
Age (y)	68.9 ± 8.2	
Race		
White	31	
Black	4	
Other	7	
Unknown	5	
Ethnicity		
Hispanic	2	
Non-Hispanic	45	
	Baseline	Follow up
PSA (ng/mL)	4.4 ± 2.5	4.5 ± 2.4
PSAD * (ng/mL/mL, mean ± SD)	0.08 ± 0.03	0.07 ± 0.03
Patient level biopsy results	N = 47	N = 17
GGG 1	46	11
GGG 2	1	4
Negative biopsy	0	2
Number of lesions ^	N = 37 lesions in 31 patients	
Lesion mean size, SD, and range (cm)	0.86 ± 0.37, 0.20–1.70	0.87 ± 0.39, 0.20–1.50
Lesion location		
Peripheral zone	28	28
Transition zone	9	9
Lesion classification ^		
PI-RADS 2 lesions	3	5
PI-RADS 3 lesions	12	12
PI-RADS 4 lesions	21	19
PI-RADS 5 lesions	1	1

* PSAD: defined as PSA/prostate volume calculated with MRI; ^ Two lesions were reclassified based on PI-RADS score at the follow up MRI from PI-RADS 4 to PI-RADS 2. 16 patients had PI-RADS 2 at MRI. Abbreviations: PSAD: prostate specific antigen density; GGG: Gleason grade group; PI-RADS: Prostate Imaging Reporting and Data System; SD: Standard Deviation.

**Table 2:** Intra-platform reproducibility (test–retest) assessment of radiomics features in prostate lesions, peripheral zone (PZ) and transition zone (TZ) using interclass correlation coefficient (ICC) for 37 patients who were scanned on the same scanner at baseline and follow-up. Overall, the proportion of ICCs that were good (ICC ≥ 0.8) or moderate (ICC ≥ 0.5) was highest on T2-WI, with a greater proportion of radiomics features with poor ICCs (<0.5) extracted from DWI and ADC. The median and interquartile ranges [IQRs] of the test–retest CoV are shown for each sequence for lesion, PZ, and TZ.

	T2W	DWI	ADC
Lesion			
Good	23 (21.2%)	2 (1.8%)	2 (1.8%)
Moderate	61 (56.4%)	18 (16.6%)	26 (24.0%)
Poor	24 (22.2%)	88 (81.4%)	80 (74%)
CV	0.136 [0.082, 0.192]	0.244 [0.120, 0.346]	0.201 [0.102, 0.289]
Peripheral Zone			
Good	17(15.7%)	5 (4.6%)	8 (7.4%)
Moderate	34 (31.4%)	9 (8.3%)	32 (29.6%)
Poor	57 (52.7%)	94 (87%)	68 (62.9%)
	0.068 [0.027, 0.114]	0.133 [0.060, 0.228]	0.120 [0.052, 0.181]
Transition Zone			
Good	25 (23.1%)	12 (11.1%)	30 (27.7%)
Moderate	63 (58.3%)	10 (9.2%)	18 (16.6%)
Poor	20 (18.5%)	86 (79.6%)	60 (55.5%)
CV	0.053 [0.028, 0.085]	0.113 [0.054, 0.182]	0.097 [0.048, 0.167]

Intraclass correlation coefficients (ICCs) were defined as good (ICC ≥ 0.8), moderate (0.5–0.8), or poor (<0.5).

**Table 3:** Inter-platform reproducibility assessment of radiomics features in prostate lesions, background peripheral zone (PZ), and transition zone (TZ) using interclass correlation coefficient (ICC) for 10 patients who were scanned on different 3T MRI systems at baseline and follow-up. Overall, the proportion of ICCs that were good (ICC ≥ 0.8) or moderate (ICC ≥ 0.5) was highest on the T2-WI, with a greater proportion of radiomics features with poor ICCs (<0.5) extracted from the DWI and ADC. The median and interquartile range [IQRs] of the test–retest CoV are shown for each sequence for lesion, PZ, and TZ.

	T2W	DWI	ADC
Lesion			
Good	33 (30.5%)	9 (8.3%)	7 (6.4%)
Moderate	28 (25.9%)	11 (10.1%)	20 (18.5%)
Poor	47 (43.5%)	88 (81.4%)	81 (75%)
	0.107 [0.050, 0.206]	0.178 [0.108, 0.285]	0.180 [0.103, 0.283]
Peripheral Zone			
Good	17 (15.7%)	6 (5.5%)	6 (5.5%)
Moderate	48 (44.4%)	7 (6.4%)	20 (18.5%)
Poor	43 (39.8%)	95 (87.9%)	82 (75.9%)
CV	0.069 [0.031, 0.119]	0.164 [0.079, 0.268]	0.144 [0.066, 0.262]
Transition Zone			
Good	5 (4.6%)	3 (2.7%)	3 (2.7%)
Moderate	41 (37.9%)	12 (11.1%)	24 (22.2%)
Poor	62 (57.4%)	93 (86.1%)	81 (75%)
CV	0.085 [0.045, 0.117]	0.161 [0.073, 0.287]	0.113 [0.060, 0.211]

Intraclass correlation coefficients (ICCs) were defined as good (ICC ≥ 0.8), moderate (0.5–0.8), or poor (<0.5).

**Table 4:** Inter-observer reproducibility assessment of radiomics measurement stability in prostate lesions, background peripheral zone (PZ) and transition zone (TZ) using interclass correlation coefficient (ICC) for patients who were scanned on different 3T MRI systems at baseline and follow-up. Overall, most features yielded good/moderate ICCs (≥0.5) for all sequences and regions.

	T2W	DWI	ADC
Lesion			
Good	20 (18.5%)	22 (20.3%)	57 (52.7%)
Moderate	42 (38.8%)	42 (38.8%)	37 (34.2%)
Poor	46 (42.5%)	44 (40.7%)	14 (13%)
Peripheral Zone			
Good	26 (24%)	59 (54.6%)	51 (47.2%)
Moderate	53 (49%)	17 (15.7%)	37 (34.2%)
Poor	29 (26.8%)	32 (29.6%)	20 (18.5%)
Transition Zone			
Good	50 (46.3%)	74 (68.5%)	53 (49%)
Moderate	50 (46.3%)	22 (20.3%)	26 (24.0%)
Poor	8 (7.4%)	12 (11.1%)	29 (26.8%)

Intraclass correlation coefficients (ICCs) were defined as good (ICC ≥ 0.8), moderate (0.5–0.8), or poor (<0.5).

**Table 5:** Comparison of the proportion of radiomics features with good/moderate vs. poor ICC as stratified by clinical and imaging variables. There was no influence of patient BMI (using a threshold of BMI = 30) on the reproducibility of prostate lesion radiomics measurements, while there was an influence for measurements in the background TZ for both the intra-platform and inter-platform evaluations and PZ for inter-platform. Aside from T2-WI on the intra-platform analysis and ADC on the inter-platform analysis, prostate volume based on median threshold volume of 63.3 mL in our study population did not influence the reproducibility of radiomics in prostate lesions. There was an influence of prostate volume on T2-W measurements in the PZ and TZ and DWI in the TZ on the intra-platform analysis. *p*-values from Fisher’s exact test are shown in the table below, and values that were significant are in bold.

BMI (≥30 vs. <30)				Prostate Volume (≥63.3 mL vs. <63.3 mL)		
Intra-platform				Intra-platform		
	ADC	DWI	T2	ADC	DWI	T2
Lesion	0.365	0.49	0.61	0.71	0.55	**0.0002**
PZ	0.999	0.099	0.66	0.54	0.086	**0.014**
TZ	**0.0068**	0.068	**0.0175**	0.37	**0.044**	**0.005**
Inter-platform				Inter-platform		
Lesion	0.051	0.218	0.999	**0.0042**	0.12	0.56
PZ	**0.004**	**0.0036**	**0.0094**	0.999	0.34	0.152
TZ	**0.0037**	**0.027**	**0.032**	0.76	0.41	0.51

## Data Availability

The raw data supporting the conclusions of this article will be made available by the authors on request.

## Supplementary Materials

The following supporting information can be downloaded at: https://www.mdpi.com/article/10.3390/cancers18050778/s1, Table S1. 3T bpMRI protocol with the sequence parameters for axial T2WI and DWI for both MRI platforms (Platform 1: Siemens Skyra; Platform 2: GE Discovery 750) are shown below. Figure S1. The radiomics analysis pipeline is shown below.

## Abbreviations

The following abbreviations are used in this manuscript:

AS	Active surveillance
ADC	Apparent diffusion coefficient
CV	Coefficient of variation
DWI	Diffusion-weighted imaging
ICC	Intraclass correlation coefficient
MRI	Magnetic resonance imaging
PCa	Prostate cancer
PSA	Prostate-specific antigen
PSAD	Prostate-specific antigen density
T1-WI	T1-weighted imaging
T2-WI	T2-weighted imaging
