# Supplementary material for: Reproducibility of MRI Radiomics Measurements in Men with Prostate Cancer Undergoing Active Surveillance
Source: Cancers (Basel). 2026 Feb 28;18(5):778. doi: 10.3390/cancers18050778 (PMC12984117; doi:10.3390/cancers18050778)

Supplementary Table S1. 3T bpMRI protocol with the sequence parameters for axial T2WI and DWI for both MRI platforms (Platform 1: Siemens Skyra; Platform 2: GE Discovery 750) are shown below.

Platform	Sequence	TR	TE	FA	SA	GRAPPA	AT	ST	FOV	Matrix
1	Axial T2WI	5610	121	137	2	2	4:42	3	180x180	320x320
2	Axial T2WI	4318	135	111	3	2	5:00	3	180x180	320x256
1	Axial DWI*	10500	min	90	1,4,8	2	7:44	3	220x220	100x100
2	Axial DWI^	5085	59.5	90	4, 14, 14	2	8:22	3	240x240	128 x 64

Abbreviations: TR (time to repetition), TE (time to echo), FA (flip angle), SA (signal averages), GRAPPA (Generalized Autocalibrating Partial Parallel Acquisition factor), AT [Acquisition time (min:sec)], ST [slice thickness (mm)], and FOV (field of view). *b-values used were 50,1000, 1600. ^b-values used were 50,1000, 1500.

Supplemental Figure S1. The radiomics analysis pipeline is shown below.

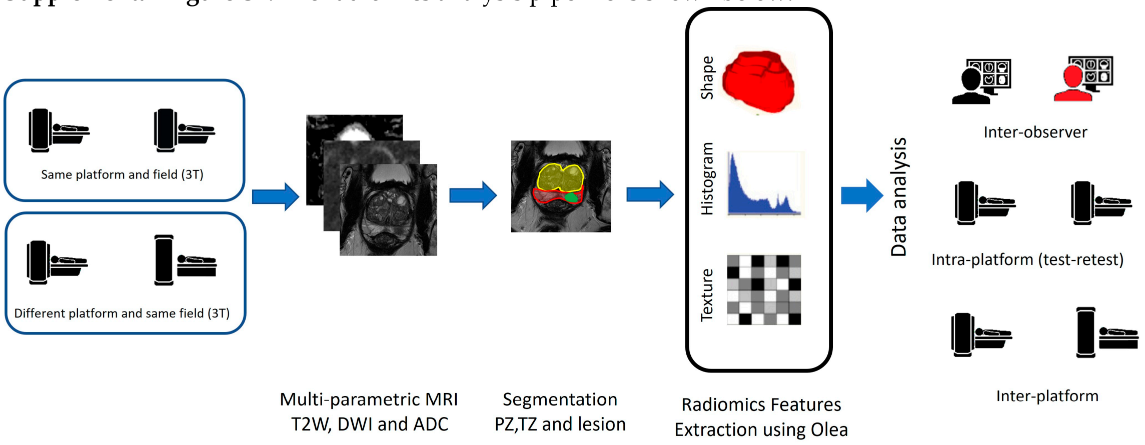

Supplement: Supplementary file 1 [file cancers-18-00778-s001.zip › cancers-4142164-supplementary.pdf]
